# Distribution of Microbial Contaminants of Minimally Processed Salads Produced in Tunisia: Need to Strengthen Good Hygiene Practices

**DOI:** 10.1155/ijfo/9570620

**Published:** 2025-02-25

**Authors:** Widad Zernadji, Faten Rahmani, Sihem Jebri, Moktar Hamdi, Manel Khammassi, Soukeina Dhib, Fatma Hmaied

**Affiliations:** ^1^Higher School of Food Industries, University of Carthage, Tunis, Tunisia; ^2^Laboratoire de Biotechnologies et Technologie Nucléaire, Centre National des Sciences et Technologies Nucléaires (CNSTN), Pôle Technologique Sidi Thabet, Sidi Thabet, Tunisia; ^3^Laboratory of Microbial Ecology and Technology (LETMI), National Institute of Applied Sciences and Technology (INSAT), Tunis, Tunisia; ^4^Research Unit of Wildlife Ecology, UR17-ES44, Faculty of Sciences of Tunis, University of Tunis El Manar, Tunis, Tunisia

**Keywords:** antibiotic resistance, food safety, norovirus, One Health, ready-to-eat salads, *Staphylococcus aureus*

## Abstract

The microbiological safety of ready-to-eat (RTE) salads is considered as a major concern due to the absence of lethal treatments during processing. In this study, we aimed to investigate the microbiological quality of RTE salads commercialized in Tunisia and to determine the antibiotic resistance of isolated pathogens, in particular *Staphylococcus aureus* (*S. aureus*). A total of 100 samples were analyzed for total aerobic bacteria, total coliforms, *Escherichia coli* (*E. coli*), yeasts and molds, *Salmonella* spp., *Listeria monocytogenes* (*L. monocytogenes*), and *S*. *aureus* as well as norovirus (NoV) GI and GII using specific standard methods described by the International Organization for Standardization (ISO). All samples presented unacceptable microbiological quality due to high concentrations of total aerobic bacteria and yeasts (> 10^6^ CFU/g) and total coliforms (> 10^4^ CFU/g). *E. coli* and molds were detected at unsatisfactory levels in 4% and 12% of samples, respectively. The pathogens *Salmonella* spp. and *L. monocytogenes* were not detected. *S. aureus* were detected at unsatisfactory levels in 6% of samples. *S. aureus* isolates were resistant to more than five antibiotic classes. Thus, RTE salads could be a vehicle of multiresistant *S. aureus*. The total prevalence of NoV GII was 2% (mean 3.81 ± 0.30 Log GC/25 g), and no NoV GI-positive samples were identified. This study showed that the microbiological quality of RTE salads commercialized in Tunisia was unacceptable, highlighting the need to ensure good agricultural and hygiene practices from farm to fork to improve the quality and safety of these products.

## 1. Introduction

Fresh fruits and vegetables are essential elements of a human healthy diet and nutrition, as they provide requisite nutrients, vitamins, antioxidants, and fiber [1, 2]. According to the World Health Organization (WHO) and the Food and Agriculture Organization (FAO), a minimum daily consumption of 400 g of fruits and vegetables is recommended to prevent certain diseases such as cancer, diabetes, and heart disease [3]. Ready-to-eat (RTE) salads are consumed raw and do not undergo further processing before consumption. They are selected, washed, peeled, cut, packaged, and preferentially stored at +4°C [4–6]. In the last few years, an increasing demand for these products has been noticed due to consumers' awareness of the benefits of eating fresh prepared foods without chemical preservatives [7–9]. In Tunisia, consumption of minimally processed products is growing, with fresh and fresh-cut vegetables becoming an essential part of the Tunisian diet [10]. Therefore, reliable good manufacturing practices (GMPs) and hazard analysis critical control point (HACCP) programs need to be applied from farm to fork to provide safe products for consumers [11]. Meanwhile, vegetables and fruits were the most commonly foodstuffs implicated in several foodborne outbreaks and associated with the most reported case patients [7, 12]. Fresh vegetables can be contaminated during processing, including packaging, transportation, storage, and distribution [10, 13, 14]. Many relevant pathogenic microorganisms are isolated from fresh vegetables and are associated with foodborne illnesses such as *Salmonella*, *Escherichia coli O157:H7*, *Shigella* spp., and *Listeria monocytogenes* [15–17]. *Staphylococcus aureus* has been considered as one of the main foodborne pathogens associated with food poisoning worldwide, caused by eating food contaminated with staphylococci enterotoxin [18]. It can contaminate many foods, especially minimally processed vegetables [19]. Besides, the dissemination of antibiotic-resistant *S. aureus* is a serious public and clinical concern in the treatment of staphylococcal infections. Hence, it is important to analyze the antibiotic resistance patterns of *S. aureus* strains isolated from RTE products [20]. Fresh produce has also been implicated in numerous foodborne viral outbreaks, mostly caused by enteric viruses [21]. Noroviruses (NoVs) are the most common cause of nonbacterial gastroenteritis outbreaks worldwide [22]. In the United States of America (USA), 1020 NoV outbreaks were reported between August 1, 2023, and March 12, 2024 [23]. These viruses are highly infectious and can induce symptoms at 10 copies or less [24]. Leafy green vegetable salad and NoV have been ranked as the third most important risk of human infection due to consumption of food of nonanimal origin in the European Union [25]. NoVs are nonenveloped viruses with a single-stranded RNA genome. They belong to the Caliciviridae family and are classified into 10 genogroups (GI–GX) and 49 genotypes [26]. Although the main route of transmission is principally from person to person, consumption of minimally processed, contaminated foods, such as berries and vegetable salads, has been widely implicated in several NoV gastroenteritis outbreaks worldwide [25]. Annually, 550 million cases of food-borne disease caused by infectious pathogens are reported, of which more than 120 million are due to NoV [27]. According to the Public Health England report, the total number of enteric viruses' outbreaks of the 2021/2022 season has exceeded the average of the previous five seasons by 35%, of which NoV outbreaks are responsible for 98%. In fact, the number of outbreaks of NoV is increasing [28]. In Tunisia, NoV was the second most frequent virus detected after rotavirus in children, with a high prevalence of the GGII.4 genotype [29]. Due to challenges related to cell culture, the ISO has recently published a horizontal method for the detection of Hepatitis A virus (HAV) and NoV in food which is based on the quantitative RT-PCR method [30, 31]. Despite the well-known microbiological risks associated with minimally processed vegetables, limited data exist in the literature about microbial quality and safety of these fresh products in Tunisia. Therefore, the objective of this study was to evaluate the microbiological quality of RTE salads commercialized in Tunisian supermarkets and to investigate the antibiotic resistance of isolated pathogens.

## 2. Material and Methods

### 2.1. Sample Collection

**Figure 1:**
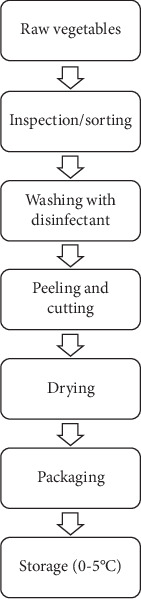
Flowchart of minimal processing steps of RTE salads.

A total of 100 samples of RTE salad were randomly collected from national supermarkets in Tunis region, Tunisia, between January 2020 and April 2022. In the supermarkets, all samples were stored in refrigerators at temperatures between 7°C and 12°C. RTE salads were packaged in plastic boxes (19 cm × 13 cm × 7 cm) made from polyethylene terephthalate (PET) material. No preservatives were used in any of the samples, and the indicated shelf life was 6 days. Samples were transferred to the laboratory in refrigerated boxes, and microbiological analyses were performed within 24 h. The salad samples were classified into four groups based on their composition (Table 1), and the manufacturing process for RTE salads is illustrated in Figure 1.

### 2.2. Microbiological Quality Analysis

#### 2.2.1. Evaluation of Bacterial, Yeast, and Mold Contamination in RTE Salads

Twenty-five grams of each sample was mixed with 225 mL of sterile medium/diluent and homogenized for 2 min in a Stomacher. Serial dilutions of the homogenates were then prepared.

For total aerobic mesophilic bacteria, 1 mL of each dilution was transferred in duplicate using Plate Count Agar medium (Biokar Diagnostics) and plates were incubated for 72 h at 30°C as described in ISO 4833-1:2013 [32]. Total coliforms were isolated according to ISO 4832:2006 [33], 1 mL of dilutions was inoculated in duplicate on Violet Red Bile Lactose Agar medium (Biokar Diagnostics) using double layer method, and plates were incubated at 37°C for 24 h. Concerning the quantification of *E. coli*, 1 mL of homogenate was inoculated in duplicate into Tryptone Bile glucuronic selective chromogenic medium agar (Biokar Diagnostics) and plates were incubated at 44°C for 18–24 h as specified in ISO 16649-2:2001 [34]. Regarding the enumeration of molds and yeasts, 100 *μ*L of each dilution was plated in duplicate into Sabouraud–chloramphenicol agar (Biokar Diagnostics) and incubated at 25°C for 3–5 days, as described in ISO 21527-1:2008 [35].

The detection of *Salmonella* spp. was carried out in accordance with ISO 6579:2002 [36]. Briefly, 1 mL and 100 *μ*L of the pre-enrichment homogenate were transferred to 10 mL of Muller Kauffmann tetrathionate broth and Rappaport Vassiliadis broth (Biokar Diagnostics) and incubated, at 37°C and 44°C, respectively, for 24 h. Subsequently, isolation was performed in duplicate using Xylose Lysine Deoxycholate agar and Hektoen enteric agar medium (Biokar Diagnostics). Plates were incubated at 37°C for 24 h. The detection of *L. monocytogenes* was performed as described in ISO 11290-2:1998 [37]. Briefly, 100 *μ*L of selective enrichment culture (25 g sample with 225 mL Fraser half broth) was added to Fraser broth (Biokar Diagnostics) and incubated for 24 h at 30°C. Then, isolation was carried out in duplicate using PALCAM agar medium (Biokar Diagnostics) and Compass Listeria agar medium (Biokar Diagnostics).

For *S. aureus* detection, 100 *μ*L of each dilution was spread onto Baird–Parker agar and the plates were incubated for 48 h at 37°C as described in ISO 6888-1 [38]. The characteristic colonies (black surrounded by light areas) were subjected to biochemical tests to confirm the presence of *S. aureus*: Gram staining, production of catalase, coagulase test. Finally, API Staph (BIOMÉRIEUX SA, France) was performed.

#### 2.2.2. Antibiotic Resistance Test

Antibiotic resistance of coagulase-positive *S. aureus* isolates was tested using the standard disk-diffusion method as reported by Li et al. [39]. Briefly, 100 *μ*L of bacteria suspension (10^7^ CFU/mL) was spread on to Mueller–Hinton Agar (Biokar Diagnostics, France) and the results were evaluated after incubation at 37°C for 24 h. The antimicrobial compounds included ampicillin (10 *μ*g), penicillin (10 IU), oxacillin (5 *μ*g), cefsulodin (30 *μ*g), ceftazidime (30 *μ*g), gentamicin (10 *μ*L), clindamycin (2 *μ*g), vancomycin (30 *μ*g), colistin (10 *μ*g), lincomycin (15 *μ*g), fusidic acid (10 *μ*g), norfloxacin (5 *μ*g), spiramycin (100 *μ*g), ticarcillin (75 *μ*g), teicoplanin (30 *μ*g), pefloxacin (5 *μ*g), imipenem (10 *μ*g), cefotaxime (30 *μ*g), ciprofloxacin (5 *μ*g), ofloxacin (5 *μ*g), meropenem (10 *μ*g), amoxicillin (25 *μ*g), piperacillin (75 *μ*L), rifampicin (30 *μ*g), and amoxicillin + clavulanic acid (20/10 *μ*g). The zone of inhibition produced by each antibiotic disc was interpreted according to Clinical and Laboratory Standards Institute zone diameter interpretation standards. *S. aureus* ATCC 25923 was used as the control strain. The antibiotic resistance was only measured for *S. aureus*, as it was the only pathogenic bacteria isolated in our sample, and we subsequently developed a nonthermal treatment to control *S. aureus* in salads.

### 2.3. Detection of NoV GI and GII

#### 2.3.1. Virus Elution and Concentration

Concentration of NOV GI and GII was performed according to the methods described in ISO/TS-15216-1 [31]. Briefly, 25 g of each sample was placed into a sterile plastic bag and homogenized with Tris glycine buffer (pH 9.5) containing 1% beef extract. The process control (mengovirus) virus particles were added and the recovery rate was 60%. The viruses were concentrated with 5x PEG/NaCl solution and then centrifuged at 10,000 *g* for 30 min at 4°C. Finally, the obtained pellet was suspended in PBS and kept frozen at −80°C, until the nucleic acid extraction and quantitative RT-PCR steps.

#### 2.3.2. RNA Extraction

Viral RNA was extracted from 200 *μ*L of sample using HigherPurity Viral RNA/DNA Extraction Kit (CANVAX, Spain) according to the manufacturer's instructions. An internal control RNA (4 *μ*L) is included in each sample from the beginning of the extraction procedure. RNA was resuspended in 50 *μ*L of RNAse-free water and stored at −20°C.

#### 2.3.3. Viral RNA Quantification by Real-Time RT-PCR

Commercial RT-qPCR kit for Norovirus GI and GII detection was used (Primer Design Ltd. Norovirus Genogroups 1 and 2 Genesig Advanced Kit, United Kingdom). The primer sequences in this kit were in accordance with those defined in the standard ISO. The RT and qPCR step occurred in the same reaction well. Internal extraction control and negative and positive controls were included in the experimental procedure according to the manufacturer's instructions. Each reaction was performed in a total volume of 20 *μ*L containing 5 *μ*L of template, 1 *μ*L of capsid or RNA-pol primer/probe mix, 1 *μ*L internal extraction control primer/probe mix, and 10 *μ*L of master mix (Oasig Lyophilized One Step 2X RT-QPCR Master Mix, United Kingdom). PCR amplification was performed on MiniOpticon System (Bio-Rad, California, United States) using the following conditions: reverse transcription at 55°C for 10 min, enzyme activation at 95°C for 2 min, followed by 50 cycles of denaturation at 95°C for 10 s and annealing at 60°C for 1 min.

### 2.4. Statistical Analysis

Microbial counts were analyzed in log scale (Log10 colony-forming unit/gram). Descriptive tests and graphics generation were performed with the SPSS software (IBM SPSS Statistics). Means comparison was based on paired samples Student's *t*-test (≤ 0.05). R Studio statistical software (R.4.0.3 version) was used to generate the principal component analysis (PCA) biplot.

## 3. Results and Discussion

### 3.1. Evaluation of Bacterial, Yeast, and Mold Contamination in RTE Salads

In Tunisia, international guidelines and standards have been applied regarding microbiological criteria for RTE vegetables. Therefore, the samples were classified as satisfactory, borderline, and unsatisfactory (Table 2).

**Figure 2:**
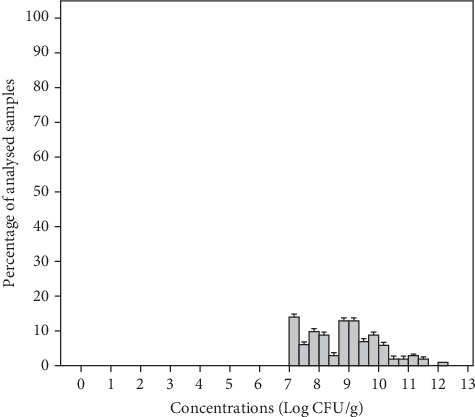
Distribution of total aerobic bacteria in the total analyzed ready-to-eat salad samples.

The present study showed that all the analyzed samples presented a total aerobic bacteria concentration higher than the acceptable limit (mean 8.82 ± 1.20 Log CFU/g) with a range of 7.22–12.00 Log CFU/g in individual samples (Figure 2). The mean concentration of total aerobic bacteria for samples analyzed in groups MS1, MS2, MS3, and MS4 was 9.59, 8.70, 8.54, and 8.57 Log CFU/g, respectively (Table 3). Thus, the highest concentrations of these micro-organisms reaching 12, 11.11, and 10.25 Log CFU/g were observed in salad samples from groups MS1, MS2, and MS3, respectively, containing grated carrots. Carrots are root vegetables, in direct contact with soil and water and therefore with manure, fertilizers, and irrigation water [42, 43]. They also undergo a series of manufacturing processes, such as peeling, slicing, and shredding before packaging which can render carrots vulnerable to cross-contamination [43]. According to microbiological guidelines (Table 2), all the salad samples (100%) were considered unacceptable for consumption. Similar results were reported by Korir et al. [44], showing high numbers of aerobic bacteria reaching 15.3 Log CFU/g in spinach and 15.28 Log CFU/g in lettuce commercialized in the United States. In another study, the number of aerobic mesophilic bacteria ranged from 7.1 to 9.5 Log CFU/g in fresh produce (lettuce, spinach) at retail levels in Korea [45]. In Portugal, 86% of RTE salad vegetables were of unsatisfactory quality due to high levels of these germs [40]. However, total aerobic bacterial concentrations in our samples were higher than those of other surveys, which ranged from 6.9 to 7.3 Log CFU/g in RTE salads commercialized in Portugal [1], from 3.50 to 7.88 Log CFU/g in RTE vegetables (parsley, lettuce, radish) marketed in the Middle East [13], from 4.3 to 8.9 Log CFU/g in minimally processed vegetables in Spain [43], and from 3 to 9 Log CFU/g in RTE coleslaw in Nigeria [42]. This might be due to the fact that all RTE included in this study are stored in supermarkets at temperatures superior to the recommended (7°C–12°C), highlighting the importance of cold food chain. In fact, these products can be subject to high microbial growth rates at temperatures above 7°C, reducing their shelf life and enhancing their microbiological risk [11]. The high levels of microorganisms in our samples may also indicate that cleaning, disinfection, or temperature control during processing, transportation, and storage could be poorly performed. Although total aerobic bacteria are not related to the safety of the product, it is one of the microbiological indicators for food quality. A high number of these microorganisms suggest contamination of the samples and generally favorable conditions for their multiplication [42, 46].

**Figure 3:**
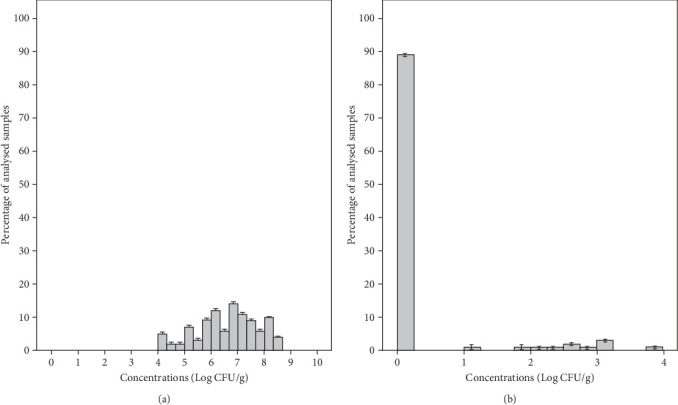
Distribution of total coliforms (a) and *Escherichia coli* (b) in the total analyzed ready-to-eat salad samples.

The mean concentration of total coliforms in the tested RTE salad samples was 6.63 ± 1.15 Log CFU/g with a range of 4–8.57 Log CFU/g in individual samples (Figure 3(a)). Meanwhile, the mean concentration of these bacteria in group salad samples MS1, MS2, MS3, and MS4 was 7.78, 6.00, 6.09, and 6.85 Log CFU/g, respectively (Table 3). Therefore, all packaged salads were classified as unsatisfactory microbiological quality due to the presence of > 10^4^ CFU/g total coliforms (Table 2). *E. coli* was detected in 11% of the samples with a mean concentration of 2.61 ± 0.71 Log CFU/g and a range of 1.12–3.80 Log CFU/g in individual samples (Figure 3(b)). The samples from groups MS1, MS2, MS3, and MS4 showed mean concentrations of 3.15, 2.64, 2.54, and 1.62 Log CFU/g, respectively (Table 3). According to microbiological guidelines (Table 2), 7% of the samples were classified as borderline quality and 4% as unsatisfactory microbiological quality. However, this germ was not detected in 89% of salads. The high number of total coliforms found in the samples as well as *E. coli* could be related to hygiene issue during preparation by the food handle or even by the use of contaminated water, since *E. coli* is a better indicator of fecal contamination during the manufacturing process, considered as a process hygiene indicator for this kind of product [40]. Similar results were reported by Tango et al. [45], showing that total coliform counts in the lettuce varied from 2.2 to 7.9 Log CFU/g. Also, total coliform counts varied from 3.5 ± 1.0 to 6.0 ± 1.1 Log CFU/g in minimally processed vegetables collected from supermarkets in Brazil [8]. Moreover, fresh-cut vegetables collected in Pakistan showed mean coliform counts of 8.0 Log CFU/g [47]. *E. coli* was detected at unsatisfactory levels in 4% of RTE salad samples collected in England [48] in 17, 6% of ready-to-use vegetable salads in Romania [9], and in 18% of minimally processed vegetables in Brazil [8].

**Figure 4:**
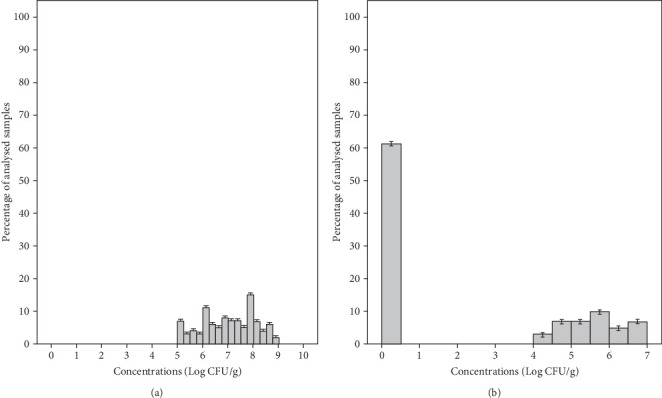
Distribution of yeasts (a) and molds (b) in the total analyzed ready-to-eat salad samples.

Yeasts were the predominant organisms and were found in all samples tested with a mean concentration of 7.03 ± 1.06 Log CFU/g and a range of 5–8.75 Log CFU/g (Figure 4(a)). Meanwhile, the mean concentration of yeasts in group salad samples MS1, MS2, MS3, and MS4 was 8.19, 7.43, 6.45, and 6.07 Log CFU/g, respectively (Table 3). Molds were found in 39% of samples, with a mean concentration of 5.60 ± 0.82 Log CFU/g and a range of 4.01–6.61 Log CFU/g (Figure 4(b)). The samples from groups MS1, MS2, MS3, and MS4 showed mean concentrations of 6.53, 5.69, 4.75, and 5.22 Log CFU/g, respectively (Table 3). In our study, the mean yeast counts (7.03 ± 1.06 Log CFU/g) were significantly higher than the mean mold counts (5.60 ± 0.82 Log CFU/g) (*p* < 0.05). In fact, minimally processed vegetables are less susceptible to molds due to the intrinsic properties of these products, such as a mildly acid to neutral pH favoring bacteria and yeasts which will overgrow molds [49]. In this study, 83% of packaged RTE salads were classified as unsatisfactory microbiological quality as they showed unacceptable levels of yeasts and only 17% were considered borderline quality. Molds were detected at satisfactory levels in 61% of the samples, 27% showed borderline levels, and 12% showed unsatisfactory levels (Table 2). Likewise, yeasts were the most prevalent detected organisms in RTE salads (such as lettuce, coleslaw, celery chunks, and baby carrots) and salad bar items (including broccoli, cauliflower, iceberg and romaine lettuce, spinach, sliced green peppers, cucumbers, and tomatoes), with concentration ranging from 2 to 6.96 Log CFU/g, while mold levels ranged from less than 2 to 3.77 Log CFU/g [50]. Moreover, the range in which this microbial group was found in other studies was 0 and 9 Log CFU/g in RTE coleslaw marketed in Nigeria [42], 3.8–7.8 Log CFU/g in mixed salads in Spain [43], and nondetected to 7.9 Log CFU/g in fresh vegetables (lettuce and spinach) in Korea [45].

The high concentrations of yeasts and molds found in this study highlight a poor hygienic quality and short shelf life for the product and this could be related to highly contaminated raw materials, poor hygiene practices during production, and improper storage conditions [17, 50]. Molds are particularly considered a health hazard for the consumers because of their ability to produce mycotoxins. They must therefore be taken into account and added to the sampling plans of the general hygiene monitoring [4, 42].

The pathogens *Salmonella* spp. and *L. monocytogenes* were not detected in all salad samples. This is in accordance with results of several studies [4, 17, 40]. However, *Salmonella* was found in 0.8% of mixed salad samples tested in Spain [43]. In Brazil, the prevalence of *Salmonella* in conventional and organic vegetables was 8.5% [51]. Furthermore, *Salmonella* was found in only one sample of ready-to-use vegetable salads (4.90 Log CFU/g) in Romania [9]. *L. monocytogenes* was detected at borderline levels in 0.2% of RTE salad samples collected in England [48]. In another study, *L. monocytogenes* was detected at unacceptable levels in one sample of RTE salad vegetables in the United Kingdom [52]. In addition, 30.5% of RTE coleslaw samples commercialized in Nigeria were positive for *L. monocytogenes* [42]. Soil, animal manure, and plant debris are potential sources of contamination of fresh produce by *L. monocytogenes* and *Salmonella* spp. [53]. According to microbiological criteria mentioned in the Commission Regulation No. 1441 (Table 2), the RTE salads evaluated in this study provided satisfactory results because they were free of these microorganisms [41].

**Figure 5:**
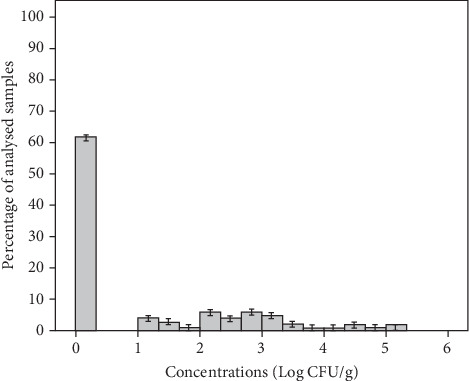
Distribution of *Staphylococcus aureus* in the total analyzed ready-to-eat salad samples.

*S. aureus* is a leading cause of food intoxication, due to its production of staphylococcal enterotoxins (SEs) [54]. RTE vegetables are processed by frequent hand contact and contain raw ingredients from multiple sources, suggesting a significant potential for *S. aureus* contamination of human and environmental origin [55]. In this study, *S. aureus* was isolated from 38% of samples, with a mean concentration of 2.77 ± 1.10 Log CFU/g and a range of 1–5.08 Log CFU/g in individual samples (Figure 5). The mean concentration of this pathogenic bacterium was 3.64, 3.29, 2.51, and 1.36 Log CFU/g in MS1, MS2, MS3, and MS4 groups, respectively (Table 3). According to microbiological guidelines (Table 2), 32% of samples were of borderline quality and 6% of unsatisfactory quality. The contamination of fresh salads with *S. aureus* can occur at different stages of the supply chain, from farm to fork. Poor hygiene practices during cultivation, harvesting, handling, and retailing can introduce these bacteria into produce [56]. In the case of minimally processed salads, *S. aureus* is a pathogen known to be carried mainly by food handlers. Nevertheless, *S. aureus* can develop in contaminated vegetables during preparation and storage under inadequate temperature conditions [57]. On the other hand, this pathogenic bacterium was not detected in 62% of samples, which indicates that good hygiene practices have been implemented by producers and processors or the use of chemical agents that could decrease the concentration of *S. aureus*. In Brazil, *S. aureus* was detected at unsatisfactory levels in 12.5% of minimally processed vegetables collected from different supermarkets [57]. The prevalence of *S. aureus* in RTE vegetables was 31.2% in China [55]. High levels of *S. aureus* were detected in RTE vegetable samples (parsley, lettuce, radish) commercialized in the Middle East reaching 6.23 Log CFU/g [13].

**Figure 6:**
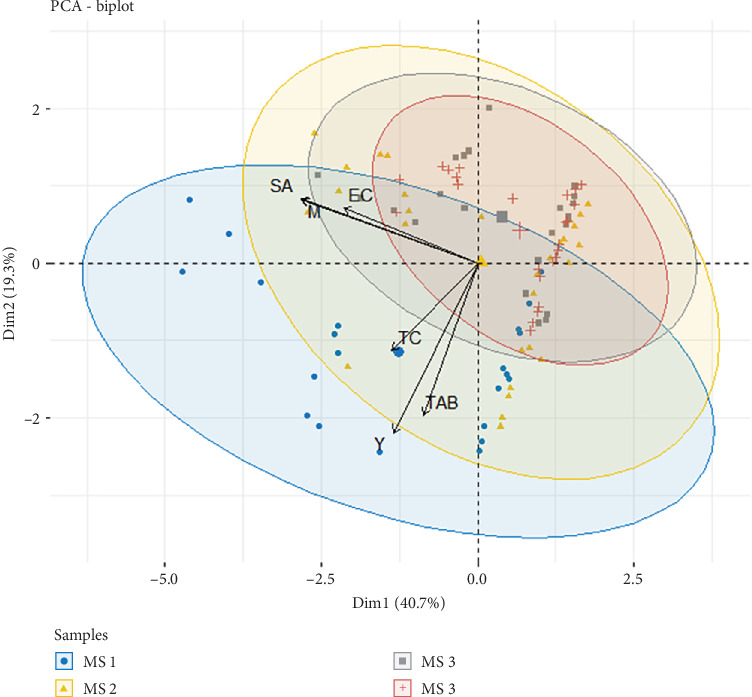
Biplot combines the scores of the ready-to-eat salad samples (data points) and the loadings of the variables on the two principal components (PC 1 and PC 2). MS, mixed salad; TC, total coliforms; TAB, total aerobic bacteria; EC, *Escherichia coli*; SA, *Staphylococcus aureus*; Y, yeasts; M, molds; MS1, lettuce, red cabbage, rocket leaves, corn, julienne carrots; MS2, lettuce, red cabbage, julienne carrots, romaine lettuce, red pepper; MS3, red lettuce, red cabbage, romaine lettuce, julienne carrots; MS4, lettuce, red pepper, green pepper, cucumber, romaine lettuce, cherry tomato.

The PCA was performed to characterize the RTE salad samples according to their composition and microbiological parameters (Figure 6). Therefore, the RTE salad samples were classified into four groups of different composition (MS1, MS2, MS3, and MS4) (Table 1). The components represented 40.7% for PC 1 and 19.3% for PC 2 of the total variance. It was found that total aerobic bacteria, total coliforms, and yeasts were positively correlated, characterizing the bacterial community of salad samples in Group MS1. Meanwhile, samples presented in Groups MS2, MS3, and MS4 were characterized by the presence of *S. aureus*, *E. coli*, and molds, which are positively correlated. The higher bacterial counts found in RTE salads can be attributed to many factors, such as irrigation water, human handling, storage containers, transport and storage temperature, and cross-contamination with other food ingredients.

### 3.2. Antibiotic Susceptibility Testing

The antibiotic susceptibility results of coagulase-positive *S. aureus* isolates from RTE salads are shown in Table 4. Overall isolates were susceptible to SP, TEC, and GM. They were resistant to TIC, PEF, IPM, CTX, CIP, OFX, MEM, AMX, PIP, RA, AMC, CM, VA, CO, L, FA, NOR, AMP, P, OX, CFS, and CAZ. The resistance of *S. aureus* isolates from different food samples has been reported elsewhere [55, 58–60]. The present study showed that *S. aureus* isolates were resistant to more than five antibiotic classes. This indicated that agricultural produce and various foods could be exposed to antibiotic-resistant strains that can cause infections in people and highlighting the One Health approach. In fact, antibiotic resistance is the most clearly illustrative of the “One Health approach” to global health concerns. It is a serious global problem that affects people, the environment, and animals. Antibiotic resistance is related to each of these three components due to the irresponsible and excessive use of antimicrobials in various sectors (agriculture, livestock, and human medicine) [61]. Our results are the first report showing that RTE salads purchased in Tunisia could be a vehicle for the transmission of multiresistant *S. aureus*.

### 3.3. Evaluation of Viral Contamination

In the present study, NoV GII was detected in two samples from Group MS1 with a mean concentration of 3.94 and 3.69 Log GC/25 g in Samples 1 and 2, respectively (Table 5), while NoV GI was not found in any analyzed sample. In fact, there are no guidelines regarding viral contamination of RTE vegetables.

The results of our study were comparable with data reported in previous investigations. The prevalence of NoV GI and GII in lettuce was 3.9% and 1.6%, respectively, in the United Kingdom [25]. The presence of NoV in fresh lettuce was analyzed by Kokkinos et al. [62], demonstrating that 2 samples were contaminated (1.3%) with NoV GI and 1 (0.8%) with NoV GII. The prevalence of NoV GII in RTE vegetables available in Italian markets was 13.6% [63]. Meanwhile, in another study, 911 samples of RTE vegetables purchased in supermarkets in Italy were analyzed and no NoV-positive samples were identified [21].

To the best of our knowledge, this is the first report documenting the presence of NoV in RTE salads in Tunisia. The detection of NoV GII in RTE salads is consistent with reported clinical studies, since NoV GII is responsible for most human NoV outbreaks in Tunisia and worldwide [29, 30]. One of the limitations of this study is the impossibility of identifying the infectious and noninfectious viral particles detected in the samples. There is no specific cell culture line for NoV in the laboratory, and its cultivation is fastidious. Photoreactive DNA-binding dye for viability PCR (PMAxx) is not available in the laboratory. Indeed, the number of genome copies detected by RT-PCR is not directly related to infectious viral particles, but the presence of viral nucleic acid revealed by these molecular assays provides a clear indication of contamination and potential risk to consumers, since a single infectious NoV has a high probability of causing infection, particularly in this type of salad which undergoes any treatment able to guarantee virus inactivation [21, 63]. Since no control measures are available to eliminate NoV without altering the characteristics of the RTE salad, the most effective risk management approach for NoV is to prevent contamination [64].

## 4. Conclusion

All analyzed samples showed unsatisfactory microbiological quality. This is the first study that has shown the presence of NoV GII in RTE salads commercialized in Tunisia, which could represent a risk for consumers. The presence of multiantibiotic-resistant *S. aureus* in RTE salads could pose public health and therapeutic concerns in consumers. Therefore, this investigation highlights the need to implement a food safety management system (FSMS) that guarantees food safety and quality throughout the supply chain. At the primary production stage, FSMS is achieved through the application of good agricultural and hygienic practices, while at the processing and commercialization stage, FSMS includes good manufacturing and hygiene practices, as well as HACCP-based principles. Distributors, retailers, and consumers must also ensure that refrigeration conditions are always maintained.

## Figures and Tables

**Table 1:** Composition and number of minimally processed salad samples.

**Symbol of salad sample groups**	**Composition**	**Number of samples**
MS1	Lettuce, red cabbage, rocket leaves, corn, julienne carrots	22
MS2	Lettuce, red cabbage, julienne carrots, romaine lettuce, red pepper	29
MS3	Red lettuce, red cabbage, romaine lettuce, julienne carrots	24
MS4	Lettuce, red pepper, green pepper, cucumber, romaine lettuce, cherry tomato	25

Abbreviation: MS, mixed salads.

**Table 2:** Microbiological limits for ready-to-eat salads.

**Microorganism**	**Contamination levels (CFU/g)**			**References**
	**Satisfactory**	**Borderline**	**Unsatisfactory**	
Total aerobic bacteria	≤ 10^4^	> 10^4^–≤ 10^6^	> 10^6^	[40]
Total coliforms	≤ 10^2^	> 10^2^–≤ 10^4^	> 10^4^	[40]
*E. coli*	≤ 10^2^	> 10^2^–< 10^3^	≥ 10^3^	[41]
*S. aureus*	< 20	20–≤ 10^4^	> 10^4^	[4]
Yeasts and molds	< 10^4^	10^4^–≤ 10^6^	> 10^6^	[4]
*L. monocytogenes*	< 10	—	> 10^2^	[41]
*Salmonella* spp.	Not detected in 25 g	—	Detected in 25 g	[41]

**Table 3:** Microbiological quality of ready-to-eat salad samples.

**Salad samples**	**Mean concentration of microbial count (Log CFU/g)**					
	**Total aerobic bacteria**	**Total coliforms**	***E. coli***	***S. aureus***	**Yeasts**	**Molds**
MS1	9.59 ± 1.13	7.78 ± 0.44	3.15 ± 0.58	3.64 ± 0.87	8.19 ± 0.60	6.53 ± 0.28
MS2	8.70 ± 1.45	6.00 ± 1.21	2.64 ± 0.29	3.29 ± 0.88	7.43 ± 0.69	5.69 ± 0.57
MS3	8.54 ± 1.12	6.09 ± 0.76	2.54 ± 0.84	2.51 ± 0.35	6.45 ± 0.53	4.75 ± 0.40
MS4	8.57 ± 0.71	6.85 ± 0.76	1.62 ± 0.70	1.36 ± 0.35	6.07 ± 0.90	5.22 ± 0.62

Abbreviation: MS, mixed salads.

**Table 4:** Antibiogram profile of *S. aureus* isolated from ready-to-eat salads.

**Antibiotic classes**	**Antibiotics**	**Sensitive/resistance**
Beta-lactams	AMP, P, OX, CAZ, CTX, TIC, AMC, AMX, CFS, IPM, MEM, PIP	R
Glycopeptides	VA	R
	TEC	S
Aminoglycosides	GM	S
Lincosamides	CM, L	R
Polymyxins	CO	R
Fusidanins	FA	R
Macrolides	SP	S
Quinolones	PEF, CIP, OFX, NOR	R
Rifamycins	RA	R

Abbreviations: AMC, amoxicillin + clavulanic acid; AMP, ampicillin; AMX, amoxicillin; CAZ, ceftazidime; CFS, cefsulodin; CIP, ciprofloxacin; CM, clindamycin; CO, colistin; CTX, cefotaxime; FA, fusidic acid; GM, gentamicin; IPM, imipenem; L, lincomycin; MEM, meropenem; NOR, norfloxacin; OFX, ofloxacin; OX, oxacillin; P, penicillin; PEF, pefloxacin; PIP, piperacillin; R, resistant; RA, rifampicin; S, sensitive; SP, spiramycin; TEC, teicoplanin; TIC, ticarcillin; VA, vancomycin.

**Table 5:** Prevalence of norovirus in ready-to-eat salad samples.

**Salad sample groups**	**Mean concentration of NoV GII (Log GC/25 g)**	
	**Sample 1**	**Sample 2**
MS1	3.94 ± 0.26	3.69 ± 0.39

Abbreviation: MS, mixed salads.

## Data Availability

The data that support the findings of this study are available on request from the corresponding author. The data are not publicly available due to privacy or ethical restrictions.

## References

[1] Costa-Ribeiro A., Azinheiro S., Mota S., Prado M., Lamas A., Garrido-Maestu A. (2024). Assessment of the presence of *Acinetobacter* spp. resistant to *β*-lactams in commercial ready-to-eat salad samples. *Food Microbiology*.

[2] Balali G. I., Yar D. D., Afua dela V. G., Adjei-Kusi P. (2020). Microbial contamination, an increasing threat to the consumption of fresh fruits and vegetables in today’s world. *International Journal of Food Microbiology*.

[3] Allen K. J., Kovacevic J., Cancarevic A. (2013). Microbiological survey of imported produce available at retail across Canada. *International Journal of Food Microbiology*.

[4] Calonico C., Delfino V., Nostro A. L. (2019). Microbiological quality of ready-to-eat salads from processing plant to the consumers. *Journal of Food and Nutrition Research*.

[5] Caponigro V., Ventura M., Chiancone I., Amato L., Parente E., Piro F. (2010). Variation of microbial load and visual quality of ready-to-eat salads by vegetable type, season, processor and retailer. *Food Microbiology*.

[6] Gurler Z., Pamuk S., Yildirim Y., Ertas N. (2015). The microbiological quality of ready-to-eat salads in Turkey: a focus on *Salmonella* spp. and *Listeria monocytogenes*. *International Journal of Food Microbiology*.

[7] Castro-Ibáñez I., Gil M. I., Allende A. (2017). Ready-to-eat vegetables: current problems and potential solutions to reduce microbial risk in the production chain. *LWT - Food Science and Technology*.

[8] de Aragão Freire Ferreira Finger J., de Almeida Silva G., Bernardino M. C., Andrade D. K. A., Maffei D. F., Pinto U. M. (2024). Investigating processing practices and microbiological quality of minimally processed vegetables in Brazil. *Brazilian Journal of Microbiology*.

[9] Albu E., Prisacaru A. E., Ghinea C., Ursachi F., Apostol L. C. (2024). Ready-to-use vegetable salads: physicochemical and microbiological evaluation. *Applied Sciences*.

[10] Rahmani F., Yahya M., Jebri S. (2021). Effect of gamma irradiation on microbial quality of minimally processed product in Tunisia: a case of ready to eat salad. *Journal of Bacteriology and Mycology*.

[11] Camino Feltes M. M., Arisseto-Bragotto A. P., Block J. M. (2017). Food quality, food-borne diseases, and food safety in the Brazilian food industry. *Food Quality and Safety*.

[12] Oteiza J. M., Prez V. E., Pereyra D. (2022). Occurrence of norovirus, rotavirus, hepatitis A virus, and enterovirus in berries in Argentina. *Food and Environmental Virology*.

[13] Faour-Klingbeil D., Murtada M., Kuri V., Todd E. C. D. (2016). Understanding the routes of contamination of ready-to-eat vegetables in the Middle East. *Food Control*.

[14] Singla G., Chaturvedi K., Sandhu P. P. (2020). Status and recent trends in fresh-cut fruits and vegetables. *Fresh-Cut Fruits and Vegetables*.

[15] Azimirad M., Nadalian B., Alavifard H. (2021). Microbiological survey and occurrence of bacterial foodborne pathogens in raw and ready-to-eat green leafy vegetables marketed in Tehran, Iran. *International Journal of Hygiene and Environmental Health*.

[16] Mir S. A., Shah M. A., Mir M. M., Dar B. N., Greiner R., Roohinejad S. (2018). Microbiological contamination of ready-to-eat vegetable salads in developing countries and potential solutions in the supply chain to control microbial pathogens. *Food Control*.

[17] Schuh V., Schuh J., Fronza N. (2020). Evaluation of the microbiological quality of minimally processed vegetables. *Food Science and Technology*.

[18] Gajewska J., Zakrzewski A. J., Chajęcka-Wierzchowska W., Zadernowska A. (2023). Impact of the food-related stress conditions on the expression of enterotoxin genes among *Staphylococcus aureus*. *Pathogens*.

[19] El-Hadedy D., Abu El-Nour S. (2012). Identification of *Staphylococcus aureus* and *Escherichia coli* isolated from Egyptian food by conventional and molecular methods. *Journal, Genetic Engineering & Biotechnology*.

[20] Shahraz F., Dadkhah H., Khaksar R. (2012). Analysis of antibiotic resistance patterns and detection of mecA gene in *Staphylococcus aureus* isolated from packaged hamburger. *Meat Science*.

[21] Terio V., Bottaro M., Pavoni E. (2017). Occurrence of hepatitis A and E and norovirus GI and GII in ready-to-eat vegetables in Italy. *International Journal of Food Microbiology*.

[22] Park S. Y., Kang S., Ha S. D. (2016). Antimicrobial effects of vinegar against norovirus and *Escherichia coli* in the traditional Korean vinegared green laver (Enteromorpha intestinalis) salad during refrigerated storage. *International Journal of Food Microbiology*.

[23] Centers for Disease Control and Prevention (2024). Norovirus outbreaks reported to NoroSTAT. https://www.cdc.gov/norovirus/reporting/norostat/data.html.

[24] El-Senousy W. M., Costafreda M. I., Pintó R. M., Bosch A. (2013). Method validation for norovirus detection in naturally contaminated irrigation water and fresh produce. *International Journal of Food Microbiology*.

[25] Cook N., Williams L., D'Agostino M. (2019). Prevalence of norovirus in produce sold at retail in the United Kingdom. *Food Microbiology*.

[26] Cammarata R. V., Barrios M. E., Díaz S. M. (2021). Assessment of microbiological quality of fresh vegetables and oysters produced in Buenos Aires Province, Argentina. *Food and Environmental Virology*.

[27] Gao J., Xue L., Li Y. (2024). A systematic review and meta-analysis indicates a high risk of human noroviruses contamination in vegetable worldwide, with GI being the predominant genogroup. *International Journal of Food Microbiology*.

[28] Public Health England National norovirus and rotavirus bulletin routine norovirus and rotavirus surveillance in England, 2021 to 2022 season week 40 report: data to week 38; 2021. https://assets.publishing.service.gov.uk/government/uploads/system/uploads/attachment_data/file/1023731/UKHSA-norovirus-bulletin-2021-22-week-40.pdf.

[29] Sdiri-Loulizi K., Hassine M., Gharbi-Khelifi H. (2011). Molecular detection of genogroup I sapovirus in Tunisian children suffering from acute gastroenteritis. *Virus Genes*.

[30] Yasmin F., Ali S. H., Ullah I. (2022). Norovirus outbreak amid COVID-19 in the United Kingdom; priorities for achieving control. *Journal of Medical Virology*.

[31] ISO 15216–1:2017 (2017). Microbiology of the food chain — horizontal method for determination of hepatitis A virus and norovirus using real-time RT-PCR — part 1: method for quantification.

[32] ISO 4833–1:2013 (2013). Microbiology of the food chain – horizontal method for the enumeration of microorganisms – part 1: colony count at 30°C by the pour plate technique.

[33] ISO 4832:2006 (2006). Microbiology of food and animal feeding stuffs - horizontal method for the enumeration of coliforms - colony-count technique.

[34] ISO 16649–2:2001 (2001). Microbiology of food and animal feeding stuffs -horizontal method for the enumeration of beta-glucuronidase-positive *Escherichia coli* -part 2: colony-count technique at 44°C using 5-bromo-4-chloro-3-indolyl *β*-D-glucuronide.

[35] ISO 21527–1:2008 (2008). Microbiology of food and animal feeding stuffs — Horizontal method for the enumeration of yeasts and moulds - part 1: colony count technique in products with water activity greater than 0, 95.

[36] ISO 6579:2002 (2002). Microbiology of food and animal feeding stuffs — horizontal method for the detection of *Salmonella* spp.

[37] ISO 11290–2:1998 (1998). Microbiology of food and animal feeding stuffs — horizontal method for the detection and enumeration of *Listeria monocytogenes* — part 2: enumeration method.

[38] ISO 6888–1 (1999). Microbiology of food and animal feeding stuffs—horizontal method for enumeration of coagulase-positive staphylococci (Staphylococcus aureus and other species)—part 1: technique using Baird-Parker agar medium. https://www.iso.org/standard/76672.html.

[39] Li H., Tang T., Stegger M., Dalsgaard A., Liu T., Leisner J. J. (2021). Characterization of antimicrobial-resistant *Staphylococcus aureus* from retail foods in Beijing, China. *Food Microbiology*.

[40] Campos J., Mourão J., Pestana N., Peixe L., Novais C., Antunes P. (2013). Microbiological quality of ready-to-eat salads: an underestimated vehicle of bacteria and clinically relevant antibiotic resistance genes. *International Journal of Food Microbiology*.

[41] Commission regulation (EC) No 1441/2007 of 5 December 2007 amending regulation (EC) No 2073/2005 on microbiological criteria for foodstuffs. *Official Journal of the European Union*.

[42] Alegbeleye O., Alegbeleye I., Oroyinka M. O. (2023). Microbiological quality of ready to eat coleslaw marketed in Ibadan, Oyo-State, Nigeria. *International Journal of Food Properties*.

[43] Abadias M., Usall J., Anguera M., Solsona C., Viñas I. (2008). Microbiological quality of fresh, minimally-processed fruit and vegetables, and sprouts from retail establishments. *International Journal of Food Microbiology*.

[44] Korir R. C., Parveen S., Hashem F., Bowers J. (2016). Microbiological quality of fresh produce obtained from retail stores on the Eastern Shore of Maryland, United States of America. *Food Microbiology*.

[45] Tango C. N., Wei S., Khan I. (2018). Microbiological quality and safety of fresh fruits and vegetables at retail levels in Korea. *Journal of Food Science*.

[46] Aycicek H., Oguz U., Karci K. (2006). Determination of total aerobic and indicator bacteria on some raw eaten vegetables from wholesalers in Ankara, Turkey. *International Journal of Hygiene and Environmental Health*.

[47] Sair A. T., Masud T., Sohail A., Rafique A. (2017). Microbiological variation amongst fresh and minimally processed vegetables from retail establishers-a public health study in Pakistan. *Food Research*.

[48] McLauchlin J., Aird H., Amar C. F. L. (2022). Microbiological quality of ready-to-eat salad products collected from retail and catering settings in England during 2020 to 2021. *Journal of Food Protection*.

[49] Siroli L., Patrignani F., Serrazanetti D. I., Gardini F., Lanciotti R. (2015). Innovative strategies based on the use of bio-control agents to improve the safety, shelf-life and quality of minimally processed fruits and vegetables. *Trends in Food Science & Technology*.

[50] Tournas V. H. (2005). Moulds and yeasts in fresh and minimally processed vegetables, and sprouts. *International Journal of Food Microbiology*.

[51] Padovani N. F., Santos T. S., Almeida P. (2023). *Salmonella* and other *Enterobacteriaceae* in conventional and organic vegetables grown in Brazilian farms. *Brazilian Journal of Microbiology*.

[52] Sagoo S. K., Little C. L., Mitchell R. T. (2003). Microbiological quality of open ready-to-eat salad vegetables: effectiveness of food hygiene training of management. *Journal of Food Protection*.

[53] Maffei D. F., Batalha E. Y., Landgraf M., Schaffner D. W., Franco B. D. G. M. (2016). Microbiology of organic and conventionally grown fresh produce. *Brazilian Journal of Microbiology*.

[54] Minutillo R., Pirard B., Fatihi A. (2023). The enterotoxin gene profiles and enterotoxin production of *Staphylococcus aureus* strains isolated from artisanal cheeses in Belgium. *Food*.

[55] Jia K., Qin X., Bu X. (2024). Prevalence, antibiotic resistance and molecular characterization of Staphylococcus aureus in ready-to-eat fruits and vegetables in Shanghai, China. *Current Research in Food Science*.

[56] Habib I., Lakshmi G. B., Mohamed M. Y. I. (2024). *Staphylococcus* spp. in salad vegetables: biodiversity, antimicrobial resistance, and first identification of methicillin-resistant strains in the United Arab Emirates food supply. *Food*.

[57] da Cruz M. R. G., de Souza Leite Y. J. B., de Lima Marques J. (2019). Microbiological quality of minimally processed vegetables commercialized in Brasilia, DF, Brazil. *Food Science and Technology*.

[58] Puah S. M., Chua K., Tan J. (2016). Virulence factors and antibiotic susceptibility of *Staphylococcus aureus* isolates in ready-to-eat foods: detection of *S. aureus* contamination and a high prevalence of virulence genes. *International Journal of Environmental Research and Public Health*.

[59] Wu S., Huang J., Wu Q. (2018). Prevalence and characterization of *Staphylococcus aureus* isolated from retail vegetables in China. *Frontiers in Microbiology*.

[60] Yang X., Zhang J., Yu S. (2016). Prevalence of *Staphylococcus aureus* and methicillin-resistant *Staphylococcus aureus* in retail ready-to-eat foods in China. *Frontiers in Microbiology*.

[61] Velazquez-Meza M. E., Galarde-López M., Carrillo-Quiróz B., Alpuche-Aranda C. M. (2022). Antimicrobial resistance: one health approach. *Veterinary World*.

[62] Kokkinos P., Kozyra I., Lazic S. (2012). Harmonised investigation of the occurrence of human enteric viruses in the leafy green vegetable supply chain in three European countries. *Food and Environmental Virology*.

[63] Laura S., Irene R., Roberta B. (2012). Potential risk of norovirus infection due to the consumption of “ready to eat” food. *Food and Environmental Virology*.

[64] Torok V. A., Hodgson K. R., Jolley J., Turnbull A., McLeod C. (2019). Estimating risk associated with human norovirus and hepatitis A virus in fresh Australian leafy greens and berries at retail. *International Journal of Food Microbiology*.

